# Association between the prevalence of hyperuricemia and reproductive hormones in polycystic ovary syndrome

**DOI:** 10.1186/s12958-018-0419-x

**Published:** 2018-10-25

**Authors:** Liangshan Mu, Jiexue Pan, Lili Yang, Qianqian Chen, Ya Chen, Yili Teng, Peiyu Wang, Rong Tang, Xuefeng Huang, Xia Chen, Haiyan Yang

**Affiliations:** 1 Reproductive Medicine Center, The First Affiliated Hospital of Wenzhou Medical University, No. 96 Fuxue Road, Wenzhou, 325000 People’s Republic of China; 2 Radiology Department, The Second Affiliated Hospital and Yuying Children’s Hospital of Wenzhou Medical University, Wenzhou, 325000 People’s Republic of China; 3 Wenzhou Medical University, Wenzhou, 325000 People’s Republic of China

**Keywords:** Polycystic ovary syndrome, Uric acid, Sex hormones, Hyperuricemia

## Abstract

**Background:**

The prevalecne of hyperuricemia in polycystic ovary syndrome (PCOS) is still uncertain. We aimed to investigate the prevalence of hyperuricemia in PCOS and to determine the influence of reproductive hormones on uric acid concentration.

**Methods:**

This retrospective cross-sectional study was performed at a large reproductive medicine center. Between March 2007 and October 2016, a total of 1,183 women with PCOS and 10,772 women without PCOS were included. PCOS was diagnosed according to the Rotterdam criteria. Anthropometric parameters, blood pressure, uric acid, reproductive hormones, glucose and lipids were measured in all subjects.

**Results:**

The serum uric acid (SUA) level was higher in women with PCOS than in women without PCOS. The prevalence of hyperuricemia in women with PCOS (25.48%) was significantly higher than that in women without PCOS (8.74%). Analysis stratified for age and body mass index (BMI) showed that both the SUA level and the prevalence of hyperuricemia were higher in women with PCOS of different age and BMI groups than in women without PCOS. After adjusting for age, BMI and estimated glomerular filtration rate (eGFR), logistic regression analysis revealed that the luteinizing/follicle-stimulating hormone (LH/FSH) ratio (odds ratio (OR) = 1.20, 95% CI = 1.01–1.43) and testosterone level (OR = 1.56, 95% CI = 1.27–1.90) were positively associated with the prevalence of hyperuricemia in females with PCOS.

**Conclusions:**

The serum uric acid (SUA) level and the prevalence of hyperuricemia markedly increased in women with PCOS. The testosterone level was positively associated with the SUA level and the prevalence of hyperuricemia in females with PCOS.

## Background

Uric acid (UA) is an organic acid that is produced during purine nucleotide metabolism. Elevated serum uric acid (SUA) is closely associated with metabolic disorders, including insulin resistance [1], type 2 diabetes [2], obesity [3] and metabolic syndrome [4]. It has been reported that reproductive hormones affect the SUA level. Estradiol (E_2_) may affect the SUA level through mechanisms involving renal secretion clearance and reabsorption [5, 6]. Increased levels of E_2_ and progesterone (P) are related to a decreased UA level; in contrast, follicle-stimulating hormone (FSH) was positively associated with the UA concentration [7]. In addition, a higher UA level was correlated with an increased probability of anovulation [7]. The influence of various endocrine hormone changes in women and their association with the SUA level has not been clearly elucidated.

Polycystic ovary syndrome (PCOS) is a common endocrine and metabolic disease that affects 4–18% of reproductive-age females according to different diagnostic criteria [8–10]. Many women with PCOS are characterized by disturbances in reproductive hormones, including androgen, the luteinizing/follicle-stimulating hormone (LH/FSH) ratio and estrogens [11–13]. Whether the imbalance in these hormones affects the SUA level and the prevalence of hyperuricemia in PCOS is still uncertain. Available studies evaluating the SUA level in PCOS are rare and have yielded conflicting results [14–18]. We hypothesized that these studies with small sample sizes could not reach a solid conclusion due to the heterogeneity of PCOS.

Therefore, we aim to examine the prevalence of hyperuricemia in women with PCOS and to determine the effects of reproductive hormones on the SUA concentration.

## Methods

### Study population

This retrospective cross-sectional study recruited 1,183 women with PCOS and 10,772 women without PCOS from the Reproductive Medicine Center of The First Affiliated Hospital of Wenzhou Medical University between March 2007 and October 2016. PCOS was diagnosed according to the Rotterdam criteria [19] based on the presence of two of the following three criteria: (1) polycystic ovaries, (2) oligoamenorrhea and (3) hyperandrogenism (biochemical or clinical), with the exclusion of other endocrine disorders. No subject used medications that affect reproductive function and metabolic function within the three months preceding enrollment. The ethics committee of The First Affiliated Hospital of Wenzhou Medical University approved this study protocol, and informed consent was obtained from each individual. The study protocol conforms to the ethical guidelines of the 1975 Declaration of Helsinki as reflected in a priori approval by the institution’s human research committee.

### Measurements

Experienced nurses measured body weight and height according to standard protocol. BMI was calculated as the body weight in kilograms divided by the body height in meters squared. Blood pressure was measured in the seated position after at least 5 min of rest. All subjects received blood testing during days 2–5 of the menstrual cycle for reproductive hormones, glucose, lipids and uric acid in the morning after an overnight fast of at least 8 h. Serum FSH, LH, E_2_ and testosterone were quantified using an autoimmunoassay analyzer [Unicel Dxl 800, Beckman Coulter, USA]. Fasting plasma glucose, serum triglycerides (TG), total cholesterol (TC), low-density lipoprotein (LDL) and high-density lipoprotein (HDL) were measured by an autoanalyzer [AU 5800, Beckman, USA].

### Definitions

BMI categories included underweight (BMI < 18.5 kg/m^2^), normal weight (18.5 ≤ BMI < 25 kg/m^2^), overweight (25 ≤ BMI < 30 kg/m^2^), and obese (BMI ≥ 30 kg/m^2^). Assessment of the estimated glomerular filtration rate (eGFR) was according to the Chronic Kidney Disease-Epidemiology Collaboration formula for Whites/others (except Blacks) [20]. Hyperuricemia was defined as an SUA level of at least 6 mg/dl in women [21].

### Statistical analysis

Continuous variables are expressed as medians (interquartile ranges), and categorical variables are presented as proportions (%). Variables with a skewed distribution were logarithmically transformed before statistical analysis. Comparisons of continuous variables and categorical variables were performed with Student’s *t* tests and χ^2^ tests, respectively. Pearson correlation analysis was used to investigate correlations between reproductive hormones and the SUA level. Logistic regression models were adopted to evaluate associations between reproductive hormones and hyperuricemia. Adjusted variables included age, BMI and eGFR. All statistical analyses were performed using SAS version 9.3 (SAS Institute, Cary, NC). A two-tailed test was applied, and a *P* value of < 0.05 was considered statistically significant.

## Results

### Baseline characteristics of the study population

The characteristics of 1,183 women with PCOS and 10,772 women without PCOS are shown in Table 1. The median level (5.16 mg/dl) of SUA and the prevalence of hyperuricemia (25.48%) were significantly higher in women with PCOS than in women without PCOS (4.52 mg/dl and 8.74%, respectively). The levels of hormones including LH, T and LH/FSH were significantly higher while FSH was lower in PCOS. No significant difference in the E_2_ level was observed between the two groups. In addition, compared with those women without PCOS, women with PCOS were younger and had higher BMI, SBP, DBP, TG, TC and LDL and lower HDL.

**Table 1:** Baseline characteristics of the study population

Variables	Non-PCOS	PCOS	*P* value
Number	10772	1813	
Age (years)	31.00 (28.00–34.00)	29.00 (27.00–31.00)	< 0.001
BMI (kg/m^2^)	21.08 (19.53–23.03)	22.51 (20.31–25.22)	< 0.001
SBP (mm Hg)	113.00 (105.00–121.00)	117.00 (108.00–125.00)	< 0.001
DBP (mm Hg)	71.00 (67.00–78.00)	74.00 (70.00–80.00)	< 0.001
FSH (IU/L)	7.70 (6.53–9.16)	6.64 (5.61–7.72)	< 0.001
LH (IU/L)	4.37 (3.29–5.79)	6.51 (4.64–8.80)	< 0.001
LH/FSH	0.55 (0.41–0.75)	0.98 (0.70–1.35)	< 0.001
E2 (pmol/L)	144.00 (101.00–190.25)	141.00 (98.00–195.00)	0.12
T (nmol/L)	1.21 (0.89–1.55)	1.65 (1.27–2.07)	< 0.001
FPG (mmol/L)	5.30 (5.00–5.60)	5.30 (5.00–5.60)	0.07
TG (mmol/L)	0.88 (0.65–1.24)	1.17 (0.81–1.78)	< 0.001
TC (mmol/L)	4.38 (3.90–4.90)	4.57 (4.07–5.16)	< 0.001
LDL (mmol/L)	2.39 (2.02–2.83)	2.58 (2.16–3.07)	< 0.001
HDL (mmol/L)	1.44 (1.25–1.66)	1.34 (1.15–1.59)	< 0.001
eGFR (mL/min/1.73m^2^)	125.97 (120.18–129.68)	126.79 (122.43–131.33)	< 0.001
Uric acid (mg/dL)	4.52 (3.95–5.16)	5.16 (4.40–6.02)	< 0.001
Hyperuricemia (%)	8.74%	25.48%	< 0.001

The data are presented as the medians (interquartile ranges) for skewed variables or as proportions for categorical variables

### Serum uric acid level and prevalence of hyperuricemia in women with PCOS stratified by age and BMI

**Fig. 1:**
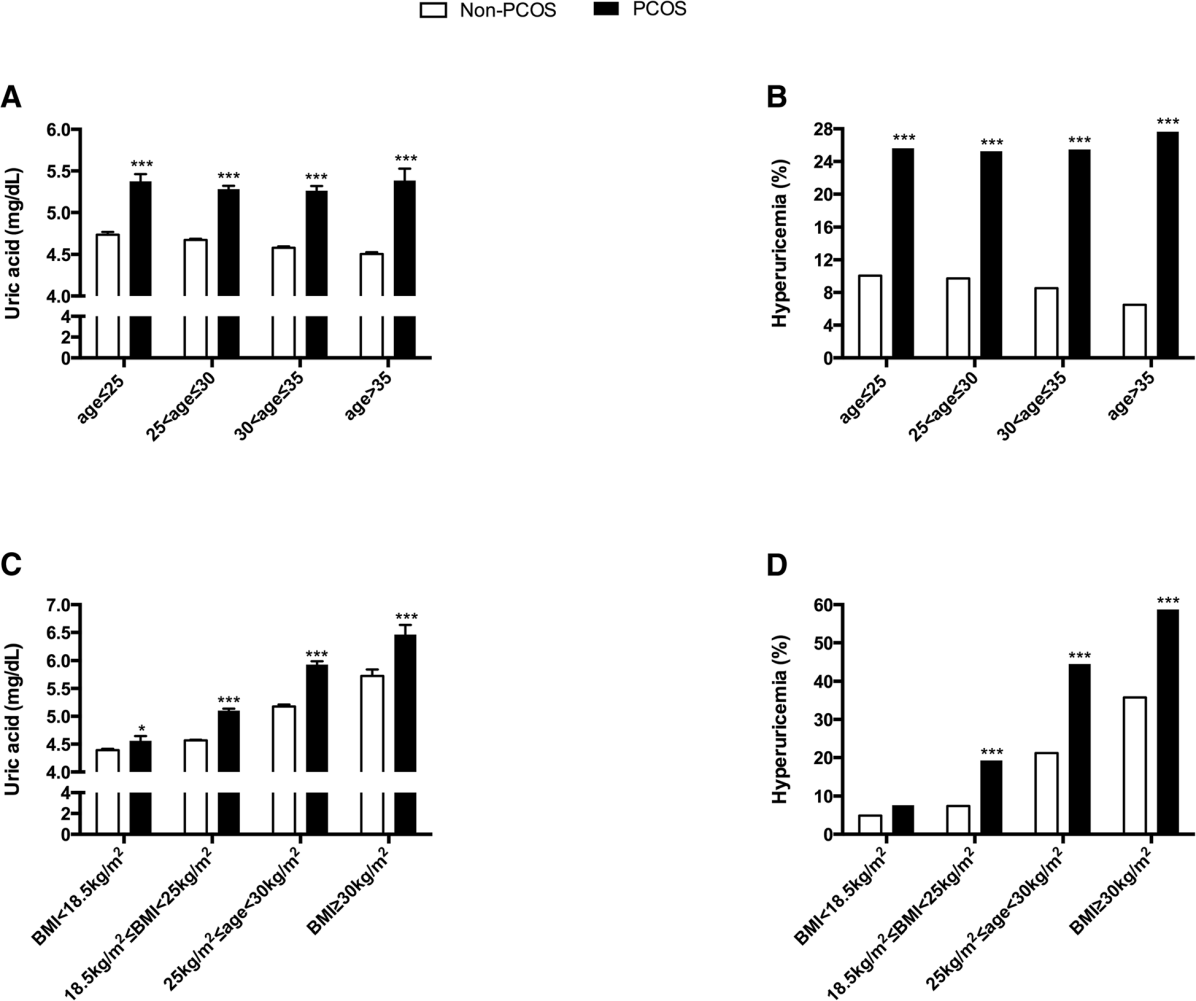
Serum uric acid level and prevalence of hyperuricemia in age and BMI-stratified women with PCOS. Uric acid levels are presented as the means+/-SEMs in A and C; the prevalences of hyperuricemia are shown in B and D. Abbreviations: PCOS, polycystic ovary syndrome; BMI, body mass index. *, *P* < 0.05; ***, *P* < 0.001

Age group analyses revealed that the mean concentrations of SUA were significantly higher in all age groups of PCOS than in non-PCOS groups (Fig. 1A). The prevalence of hyperuricemia in all age groups of patients with PCOS was greater than 25%, which was nearly twofold to fourfold higher than that in non-PCOS groups (Fig. 1B). The mean ± SEM concentrations of SUA increased from 4.56 ± 0.08 mg/dL to 6.46 ± 0.17 mg/dL with increasing BMI categories in patients with PCOS, which were significantly higher than those in the non-PCOS groups (Fig. 1C). Accordingly, the prevalence of hyperuricemia rose from 7.64 to 58.75% with increasing BMI categories in patients with PCOS. From the normal range of BMI to obesity, the prevalence of hyperuricemia in patients with PCOS was significantly higher than that in women without PCOS. The prevalence of hyperuricemia was similar between the two groups when the subjects’ BMI was less than 18.5 kg/m^2^ (Fig. 1D).

### Associations of reproductive hormones with serum uric acid and hyperuricemia in PCOS

Correlation analyses revealed that the SUA level was negatively associated with the FSH level (*r* = − 0.09, *P* < 0.001) and was positively associated with the testosterone level (*r* = 0.16, *P* < 0.001) (Table 2). Accordingly, FSH was negatively associated with hyperuricemia (OR = 0.89, 95% CI = 0.83–0.95, *P* < 0.001), and testosterone was positively associated with hyperuricemia (OR = 1.75, 95% CI = 1.45–2.11, *P* < 0.001) (Table 3). After adjusting for age, BMI and eGFR, there was still a positive correlation between the SUA and testosterone levels (*r* = 0.16, *P* < 0.001) (Table 2). In addition, both LH/FSH (OR = 1.20, 95% CI = 1.01–1.43, *P* = 0.04) and the testosterone level (OR = 1.56, 95% CI = 1.27–1.90, *P* < 0.001) were positively associated with hyperuricemia in the adjusted model (Table 3).

**Table 2:** Correlations between reproductive hormones and serum uric acid

	Unadjusted		Adjusted	
Hormones	*r*	*P*	*r*	*P*
FSH	−0.09	< 0.001	−0.03	0.17
LH	−0.03	0.16	0.004	0.88
LH/FSH	0.01	0.75	0.005	0.84
E2	−0.01	0.59	− 0.01	0.55
T	0.16	< 0.001	0.11	< 0.001

Adjusted variables include age, BMI and eGFR

*Abbreviations*: *PCOS* polycystic ovary syndrome, *FSH* follicle-stimulating hormone, *LH* luteinizing hormone, *E2* estradiol, *T* total testosterone, *BMI* body mass index, *eGFR* estimated glomerular filtration rate

**Table 3:** Associations between reproductive hormones and hyperuricemia in PCOS

	Unadjusted		Adjusted	
Hormones	OR (95%CI)	*P*	OR (95%CI)	*P*
FSH	0.89 (0.83–0.95)	< 0.001	0.94 (0.88–1.01)	0.91
LH	1.00 (0.98–1.03)	0.94	1.02 (0.99–1.05)	0.14
LH/FSH	1.14 (0.97–1.35)	0.11	1.20 (1.01–1.43)	0.04
E2	1.00 (1.00–1.00)	0.94	1.00 (1.00–1.00)	0.97
T	1.75 (1.45–2.11)	< 0.001	1.56 (1.27–1.90)	< 0.001

Adjusted variables include age, BMI and eGFR

*Abbreviations*: *PCOS* polycystic ovary syndrome, *FSH* follicle-stimulating hormone, *LH* luteinizing hormone, *E2* estradiol, *T* total testosterone, *BMI* body mass index, *eGFR* estimated glomerular filtration rate, *OR* odds ratio, *CI* confidence interval

### Discussion

In the present study, we found that both the serum uric acid level and prevalence of hyperuricemia increased in the age- and BMI-stratified PCOS population. In addition, a high level of testosterone was strongly associated with an elevated SUA level and the prevalence of hyperuricemia. To our knowledge, this study is the first to report the prevalence of hyperuricemia in such a large sample size of women with PCOS.

Previous studies analyzing the SUA level in PCOS are scarce and have yielded controversial results. Quinonez et al. and Yarali et al. found that the SUA concentration was significantly increased in women with PCOS [16, 17]. However, Anttila et al. and Manuel et al. reported that no differences in uric acid levels were detected between women with PCOS and control women [14, 15]. We hypothesize that the two main reasons for these conflicting results may be the small sample size and the BMI. The numbers of women with PCOS in these studies ranged from thirty to fifty-five. Therefore, the results from these studies may be greatly affected by the heterogeneity of PCOS. Due to the influence of obesity on the SUA level [3, 22], the different rates of obesity among the PCOS and control populations influenced these results. Therefore, we used age- and BMI-stratified analyses to further exemplify the contribution of these two key factors on the SUA level. Our present results found that the SUA level and prevalence of hyperuricemia increased greatly in both the PCOS and non-PCOS groups as BMI increased. Overall, 58.75% of women with obesity and PCOS had hyperuricemia, which was nearly threefold higher than that in women with PCOS and a normal BMI. In addition, we found that both the SUA level and the prevalence of hyperuricemia were similar in the age-stratified PCOS group. These findings indicated that elevated SUA levels in PCOS might be independent of age and BMI.

Apart from the effect of obesity on SUA level, sex hormones also play a significant part in uric acid regulation. It was found that the uric acid level varied across the menstrual cycle in healthy premenopausal women, with the highest level in the follicular phase and a decrease during the luteal phase [7]. E_2_ was inversely associated with the uric acid level [7, 23]. E_2_ may affect the serum levels of uric acid through mechanisms potentially including renal clearance, secretion and reabsorption [5, 6]. However, we did not find a correlation between E_2_ and the SUA level in PCOS. One study reported that FSH was positively associated with the uric acid concentration [7]; on the contrary, another study found a low concentration of FSH in women with gout, although it did not find a direct association between FSH and the uric acid level [24]. In our study, the negative correlation between FSH and SUA disappeared when we adjusted for age and BMI, which indicated that this association was not independent. Interestingly, we observed a positive association between LH/FSH and hyperuricemia. Thus, further mechanistic study is warranted to clarify these inconsistent findings.

Androgen excess is a common characteristic in PCOS and promotes metabolic disorders [25]. Androgens might also influence uric acid metabolism to some extent. Higher SUA concentrations in men compared with women supported a possible correlation between androgen and uric acid [26]. It was observed that free testosterone was positively associated with uric acid concentration in healthy female population [27]. Plasma metabolomics analysis found that PCOS with hyperandrogenism had higher uric acid level than PCOS with anovulation and polycystic ovaries [18]. Our results also confirmed that testosterone was positively correlated with SUA level and hyperuricemia independently of age and BMI. In women with PCOS, Diane^35^ treatments were associated with a decrease in uric acid levels, which was in parallel to the decrease in the free androgen index [15]. Animal experiments showed that androgens might increase serum uric acid levels by inducing the hepatic metabolism of purine nucleotides [28, 29] and enhancing purine turnover in the kidney [30]. These findings indicated that purine metabolism might be the target of hormonal action, although more mechanistic studies are needed to confirm this hypothesis.

Whether SUA is a cause or a risk factor, we suggest that more attention should be devoted to women with high levels of SUA, especially infertile women with obesity or/and hyperandrogenism, because uric acid is a purine derivative and purines might inhibit oocyte maturation [31, 32]. Previous evidence had demonstrated that hyperuricaemia identifies women at increased risk of adverse maternal and fetal outcome [33]. Hyperuricemia is a common finding in preeclamptic pregnancies [34]. Uric acid impacts on placental development and function and maternal vascular health through promoting inflammation, oxidative stress and endothelial dysfunction [35]. Hyperuricemia may be a pathogenic factor in preeclampsia. Further studies are needed to confirm the association between hyperuricemia and outcomes of assisted reproductive technologies. Thus, ameliorating androgen excess with antiandrogenic drugs may benefit women with hyperandrogenism partly through decreasing the SUA level. Diane^35^ treatments may help to cure hyperuricemia for PCOS women with hyperadrogenism [15].

The strength of our study is its novelty and its large number of study subjects. However, several limitations should be taken into consideration. First, these subjects are infertility patients from our center. Therefore, our results may not be generalizable to the general population. Second, considering variations in uric acid secretion in different stages of the menstrual cycle, we measured the uric acid level only during the follicular phase. Third, our study was a retrospective study based on a single center, which may introduce selection bias. In addition, our results were restricted to Chinese women. Future studies in other populations are warranted to confirm our findings.

## Conclusions

In summary, our findings confirmed that the SUA level and the prevalence of hyperuricemia markedly increased in women with PCOS independent of age and BMI. A high level of testosterone was strongly associated with an elevated SUA level and the prevalence of hyperuricemia, suggesting that androgen may be a mediator in the pathogenesis of uric acid metabolism. Future studies are warranted to clarify the potential role of androgen in the development of hyperuricemia.

## Abbreviations

BMI: Body mass index
DBP: Diastolic blood pressure
E2: Estradiol
eGFR: Estimated glomerular filtration rate
FPG: Fasting plasma glucose
FSH: Follicle-stimulating hormone
HDL: High-density lipoprotein
LDL: Low-density lipoprotein
LH: Luteinizing hormone
PCOS: Polycystic ovary syndrome
SBP: Systolic blood pressure
T: Total testosterone
TC: Total cholesterol
TG: Triglycerides
